# Management Approach for Traumatic Complex Degloving Perineal Injuries: A Retrospective Review of 6 Cases

**DOI:** 10.5152/tud.2022.21320

**Published:** 2022-03-01

**Authors:** Chin Yiun Lee, Syahril Anuar Salauddin, Hamid Ghazali

**Affiliations:** Department of Urology, Hospital Tengku Ampuan Afzan (HTAA), Kuantan, Pahang, Malaysia

**Keywords:** Degloving perineal injury, transacted urethra, retrospective review

## Abstract

**Objective:**

Degloving perineal injury is defined as avulsion of the perineal skin and subcutaneous tissue from its surrounding fascia, bone, and organs. This injury is most commonly caused by road vehicle accidents or alleged falls from heights. This devastating injury is associated with high morbidity and mortality if mismanaged. We retrospectively evaluated our management protocol for degloving perineal injury and the clinical outcomes of our case series.

**Material and Methods:**

Six cases of open perineal degloving injuries were managed and retrospectively reviewed at our center from 2018 to 2020, comprising 2 cases of isolated degloving wounds of the scrotum and 4 cases of complex perineal injuries associated with pelvic fracture, anorectal injuries, or complete transacted urethral injuries. Two of the 6 patients had open-book pelvic fractures that required pelvic stabilization.

**Results:**

Complex degloving perineal injuries can be managed through (1) resuscitation and pelvic packing; (2) urgent life-threatening treatments; (3) pelvic exploration with debridement; and (4) primary repair of anorectal or transacted urethral injuries with external pelvic fixation. All patients promptly underwent surgical repair after immediate diagnosis and resuscitation. Additionally, patients were operated by specialized teams according to the proposed management approach as shown in Figure 1. All patients recovered well and had good functional outcomes.

**Conclusion:**

A multidisciplinary team comprising general surgeons, urologists, orthopedic surgeons, and plastic surgeons was involved in this review. Prompt diagnosis and immediate surgical intervention remain the standard requirements for complex degloving perineal injuries. We proposed a management protocol that is safe for managing complex perineal injuries.

## Main Points

- A multidisciplinary team comprising general surgeons, urologists, orthopedic surgeons, and plastic surgeons should manage complex perineal injuries.
- Prompt diagnosis and immediate surgical intervention remain the standard treatments for complex perineal degloving injuries.
- All patients promptly underwent surgical repair after immediate diagnosis and resuscitation.
- All patients recovered appropriately with good functional outcomes.
- Prophylactic diversion colostomy is required to minimize the risk of postoperative wound sepsis.

## Introduction

Severe degloving injury of the perineum is a devastating consequence of trauma in road vehicle accidents. According to the Global Status Report on Road Safety, Malaysia has the third-highest incidence of road accident deaths in Asia, with a death rate of 23 per 100 000 population. Technically, the significant impact of rotational force during trauma tears the skin, subcutaneous tissues, and adjacent organ structures from the surrounding fascia. Blunt trauma contributes to approximately 8%-10% of degloving perineal injuries.^1^

Degloving perineal injury is a urological emergency that requires immediate resuscitation and surgical treatment. Immediate wound irrigation with adequate debridement of the contaminated wounds is recommended by the guidelines.^2,3^ Theoretically, when friction occurs between a moving vehicle and the skin surface of an individual, it creates a shear force that tears the surrounding soft tissues away from the surrounding structures and extends deep into the pelvic region. Consequently, this mechanism of injury can damage major vessels and cause pelvic bone fractures. The common causes of these degloving perineal injuries are direct perineal trauma in motor vehicle accidents (MVAs) or alleged accidental falls from a tree with sustained saddle injuries.

Decompensated shock secondary to uncontrolled hemorrhage deep within the degloving pelvis wound is a fatal consequence of the inability to control blood loss. Despite immediate resuscitation according to the advanced traumatic life support (ATLS) principles and packing the pelvic soft tissues, there is generally a high failure rate of controlling hemorrhage from lacerated vessels. Therefore, emergency surgery for hemostasis and reconstruction by multidisciplinary teams are required in the management of traumatic perineal injuries.

High-impact MVA injuries result in degloving perineal traumas, such as pelvic bone fracture and anorectal and urethral injuries. In addition, in spite of a patient’s survival of the initial insult from the trauma, the risk of sepsis postoperatively remains high due to wound contamination and prolonged hospitalization.^2^ Therefore, active measures of wound debridement and antibiotics administration are important to minimize the risk of sepsis and improve survival.

This study aimed to evaluate our management protocol for traumatic perineal degloving injury compared to the standard approach, with emphasis on the concept of immediate wound debridement, urinary and fecal diversion, and anastomosis of transacted urethral injury, and to evaluate the clinical outcomes of our series of 6 cases.

## Material and Methods

**Figure 1.**
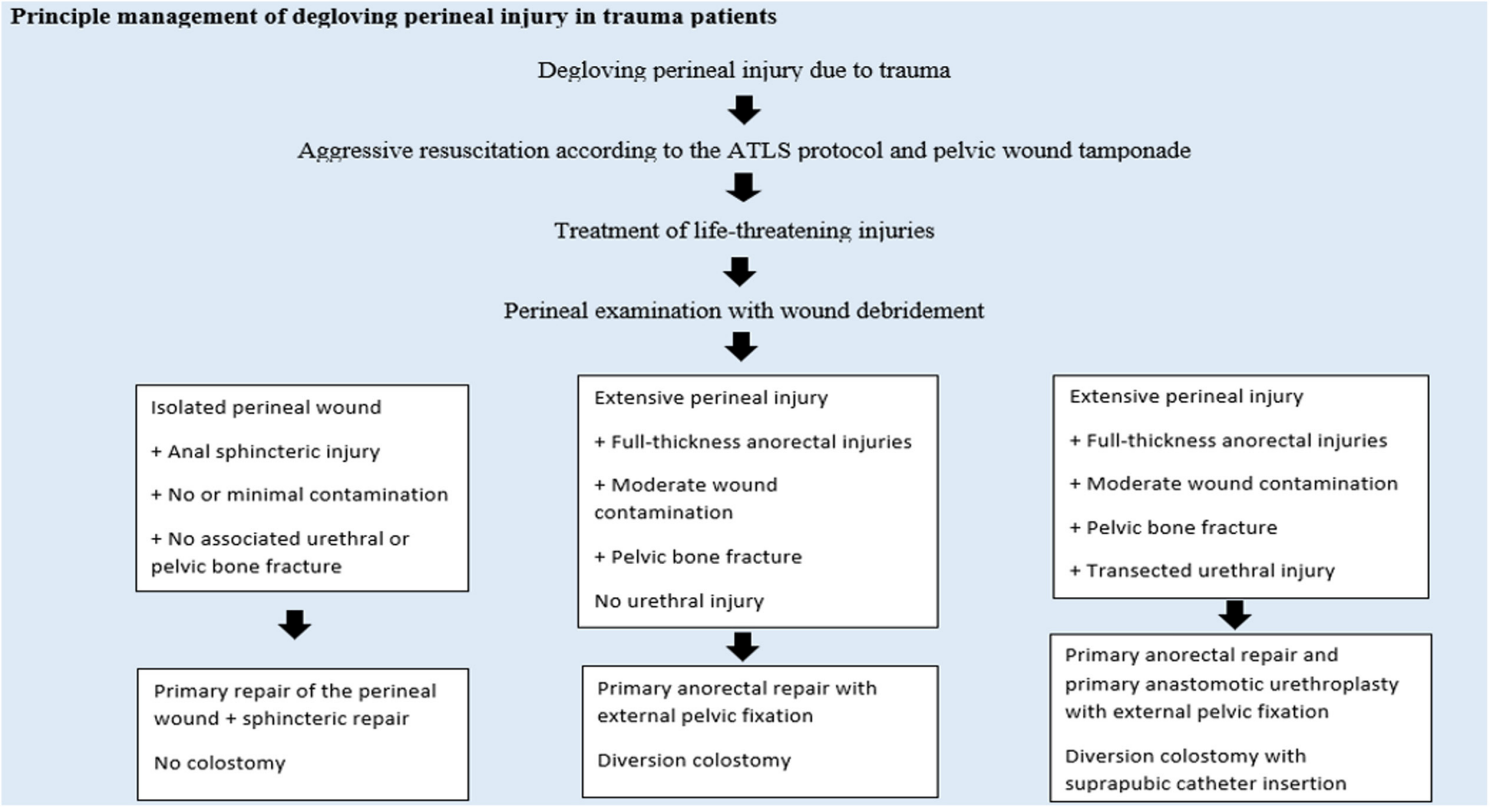
Principle management of degloving perineal injury in trauma patients.

In this study, we retrospectively analyzed 6 cases of degloving perineal injury due to trauma between 2018 and 2020. The complexity of degloving trauma with its associated organ involvement is presented in Table 1. Further, the principles of management of traumatic degloving perineal injuries are summarized in Figure 1. Etiologies of trauma, physical findings, management, postoperative infection, and functional outcomes were evaluated in all patients. The study design was exempted from ethics board review and was registered under Malaysian National Medical Research Register (NMRR ID-21-01939-3BY) and written consent was obtained from the patients for publication of this case series and accompanying image. All study participantsare age 18 and above.

## Results

A series of 6 patients with an average age of 32 years (range, 16-38 years) with serious traumatic degloving perineal injuries were evaluated. In this series, 4 patients were allegedly involved in MVAs, while 1 of the other 2 patients fell from a tree, and the other was attacked by a wild boar.

**Figure 2.**
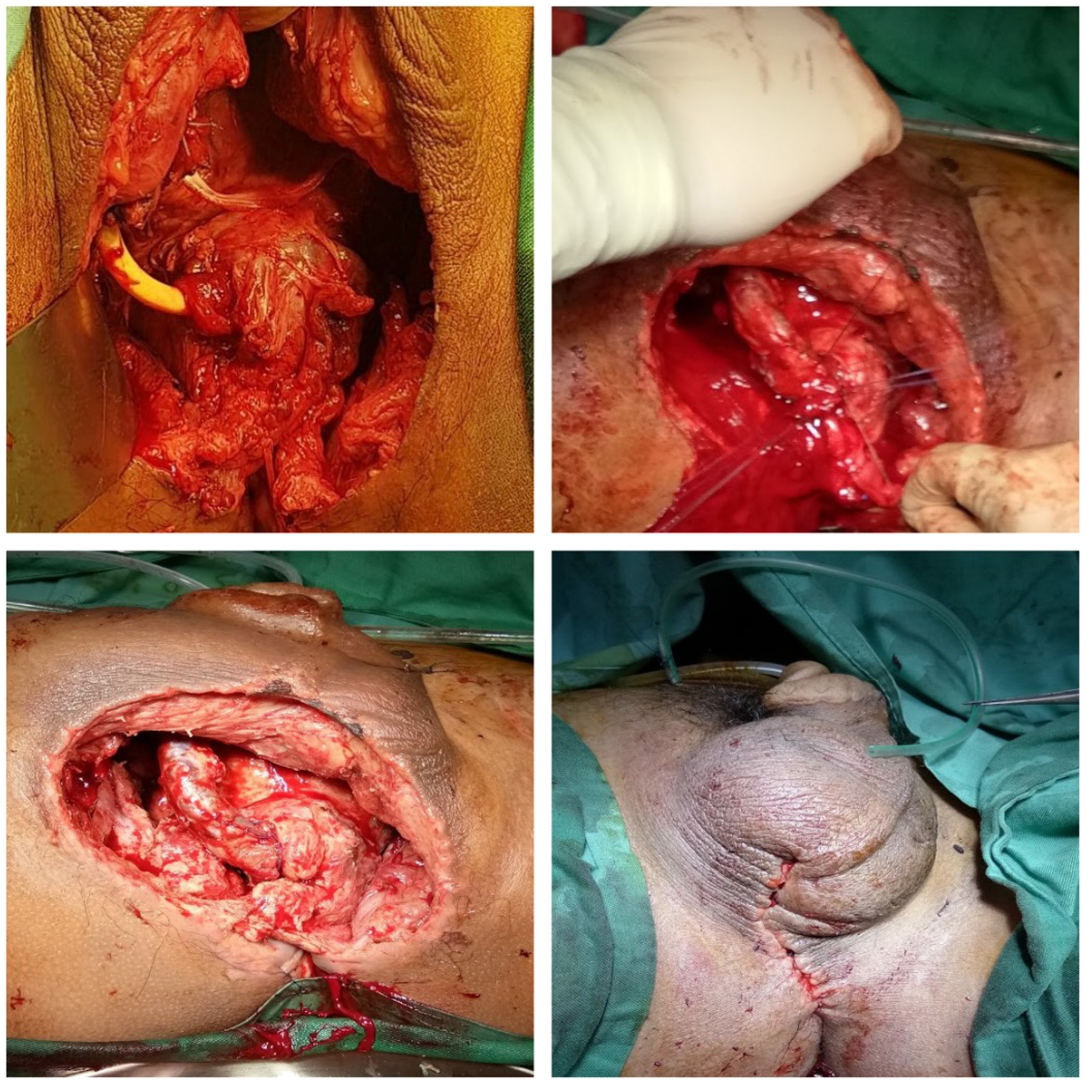
This was a case of complex degloving perineal wound associated with anorectal with sphincteric injuries and transacted bulbomembranous urethra. Primary anastomotic urethroplasty was done after radical wound debridement. Drain inserted postoperatively.

**Figure 3.**
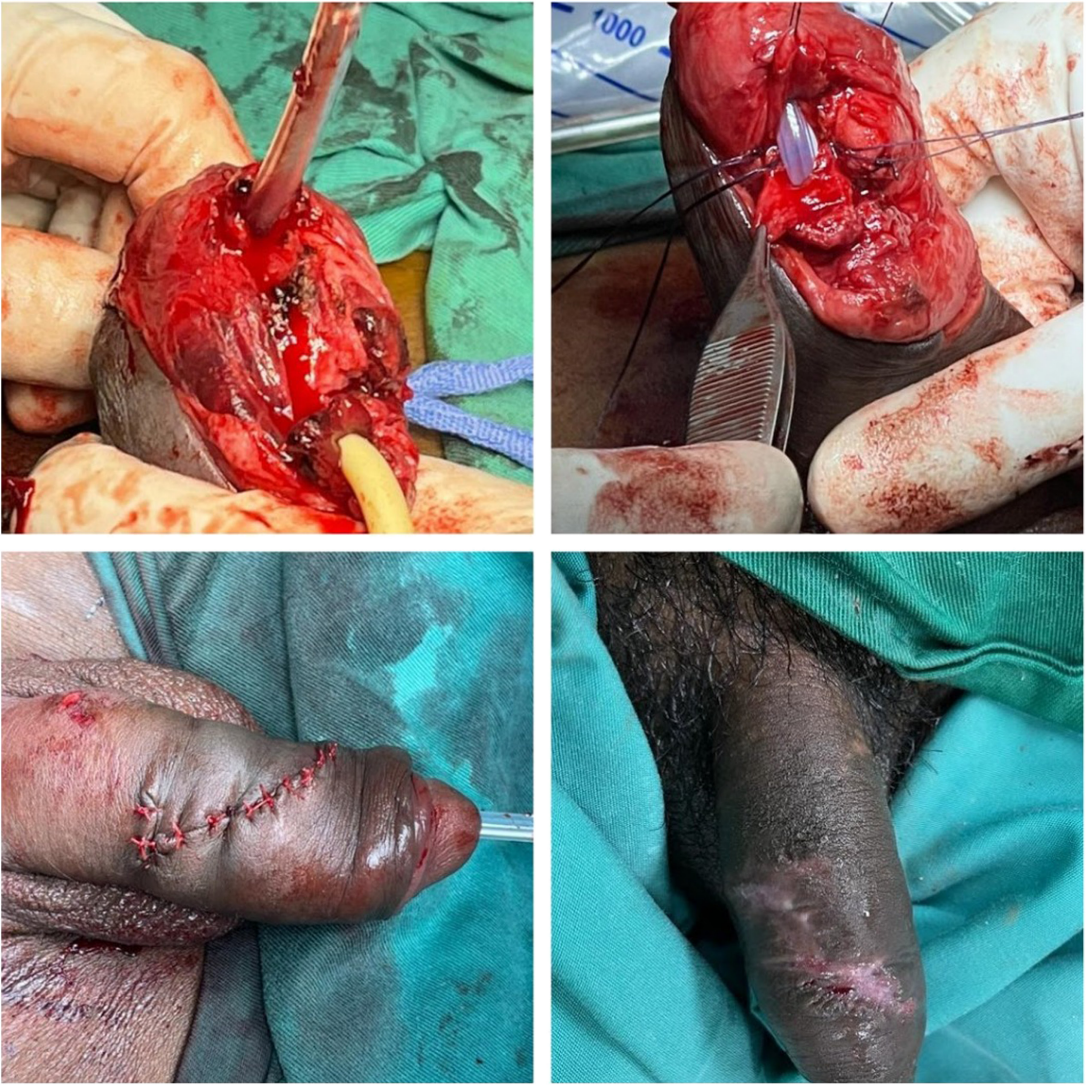
This patient sustained transacted penile urethral with the breach at tunica of corpus cavernosum. Primary repaired done and recovered well.

All patients underwent immediate resuscitation and stabilization according to the management principles of the ATLS protocol. Early blood product transfusion and perineal packing have proven to be effective approaches during resuscitation. Fortunately, all our patients survived triage and underwent urgent surgery. After the patients were sedated under general anesthesia and lifesaving operations, such as exploratory laparotomy, were performed. This was followed by pelvic exploration, saline irrigation and debridement of contaminated wounds, urinary and diversion colostomy, and primary repair of urethral and anorectal injuries. There were 2 cases of open-book pelvic fractures requiring external pelvic fixation during the initial procedure. Diversion colostomy and primary repair of anorectal injuries and anal sphincter were performed by the colorectal team for patients with full-thickness anorectal and anal sphincteric injuries. Two of the 6 patients had bladder injuries that required primary bladder repair and suprapubic catheter (SPC) insertions. Two other patients sustained transacted urethral injury in the penile and bulbomembranous regions and required immediate primary anastomotic urethroplasty as shown in Figures 2 and 3 respectively. Interestingly, a breach was observed in the tunica albuginea of the corpus cavernosum of one of the patients with a transacted urethra. An artificial erection test was performed on the table for the patient after the primary repair of the corpus cavernosum.

All patients with anorectal injuries underwent prophylactic proximal colostomies, and early urinary diversion with primary urethral anastomosis was performed in patients with sustained transacted urethral injury. None of the patients had recurrent urethral strictures during our follow-up. Prolonged hospitalization, with an average of 48 days, was recorded, and wound sepsis was the most common complication observed in this study.

Fortunately, there was no early death secondary to uncontrolled hemorrhage from the degloving perineal wounds. Although sepsis was a common complication postoperatively, all patients recovered well with conservative treatment. Most patients had a good functional outcome with a satisfactory quality of life during follow-up.

## Discussion

Without a standardized protocol for managing degloving perineal injuries, these injuries can be surgically challenging for surgeons. These complex injuries are complicated and associated with a high mortality rate if mismanaged. Hence, the first surgery is the best opportunity for patients to be managed by specialized teams to reduce comorbidity and ensure better outcomes. Limited publications on the management of severe traumatic perineal injuries and inadequate clinical experience among junior surgeons can lead to many controversies. Early death secondary to uncontrolled hemorrhage in trauma can be prevented with the early identification of hypovolemic shock and blood transfusion. In addition, activation of a massive blood transfusion protocol as necessary in trauma has been proven to be lifesaving, especially for hemodynamically unstable patients.^4^

After initial fluid resuscitation and management of life-threatening injuries, a complex degloving perineal wound should be prioritized by a clinician. Computed tomography of the abdomen and pelvis helps identify concomitant injuries of the pelvic organs, allowing an accurate diagnosis before surgery in a hemodynamically stable patient. If there is uncontrolled bleeding from the pelvis, immediate pelvic tamponade is recommended before proceeding with urgent wound exploration, especially in those with impending hypovolemic shock.

Direct perineal trauma secondary to road accidents was the primary cause of injury in this series. Perineal trauma is reportedly more common in male than in female individuals, especially among pediatric patients.^4^

During the examination of the degloving wound, all bleeding spots deep within the pelvis should be ligated and secured to provide a better view during exploration. Exploratory laparotomy is mandatory if there is evidence suggestive of intra-abdominal injuries, and broad-spectrum antibiotics should be administered to the patient prior to induction. Additionally, all devitalized fascia or heavily bruised tissue should be debrided until healthy tissue is observed. This is because tissue necrosis is caused by insufficient blood supply and potentially becomes a source of bacterial infection. In addition, inadequate debridement of contaminated wounds, residual hematoma, and fecal contamination are potential risk factors for postoperative sepsis.^3^

Management of these complex injuries by specialized teams is important because the first emergency operation ensures the best outcome and prevents serious complications. Our standard management principle is based on the severity of the degloving perineal injury and degree of wound contamination.

Two patients sustained open-book pelvic fractures together with degloving perineal injury. Initial stabilization of the pelvic ring with external fixation contributes to bleeding controlled by approximating the edges of the bone. Similarly, this procedure eases the lithotomy positioning during perineal wound exploration and minimizes the movement of the unstable pelvis and its surrounding avulsed fascia. In fact, the incidence of urethral rupture associated with pelvic fractures is reportedly approximately 70%.^5^ In our series, 2 patients with pelvic fracture had American Association for the Surgery of Trauma grade IV urethral injuries. This is because urinary structures lie adjacent to the pelvic bone, and a high-impact force can be transmitted easily from the bone to the surrounding tissues.

In managing degloving perineal injury associated with anorectal injuries, we proposed prophylactic diversion colostomy. Proctosigmoidoscopy should be performed to examine the anorectal mucosa and access the perforation. Fu et al^6^ classified perineal injuries into 3 types based on different management protocols. Additionally, they recommended initial urinary and fecal diversion based on the location, depth, and size of soft tissue defects and adjacent organ involvement.

In our study, all the patients had genitourinary injuries. Additionally, all patients underwent retrograde urethrography and cystogram to determine the severity of urethral and bladder injuries. In cases of suspicious urethral injury with urinary retention, insertion of an indwelling catheter is potentially harmful despite a single attempt; however, gentle catheterization with appropriate lubrication is permitted according to the latest European Association of Urology (EAU) guidelines. We prefer performing suprapubic catheterization under ultrasound guidance or open SPC insertion when treating acute urine retention in a trauma setting.

The acute treatment of immediate primary anastomotic urethroplasty remains controversial. According to the evidence summarized in the EAU guidelines,^7^ immediate (primary) urethroplasty is associated with a higher risk of bleeding, stricture, incontinence, and impotence as compared to delayed urethroplasty. It is proposed that this is due to further iatrogenic trauma in a setting of distorted anatomy and gross hematoma. However, early urethral re-alignment or indeed even primary urethroplasty may result in less morbidity in selected patients. Cases must be chosen very carefully so as to avoid the sequelae outlined above. 

**Figure 4.**
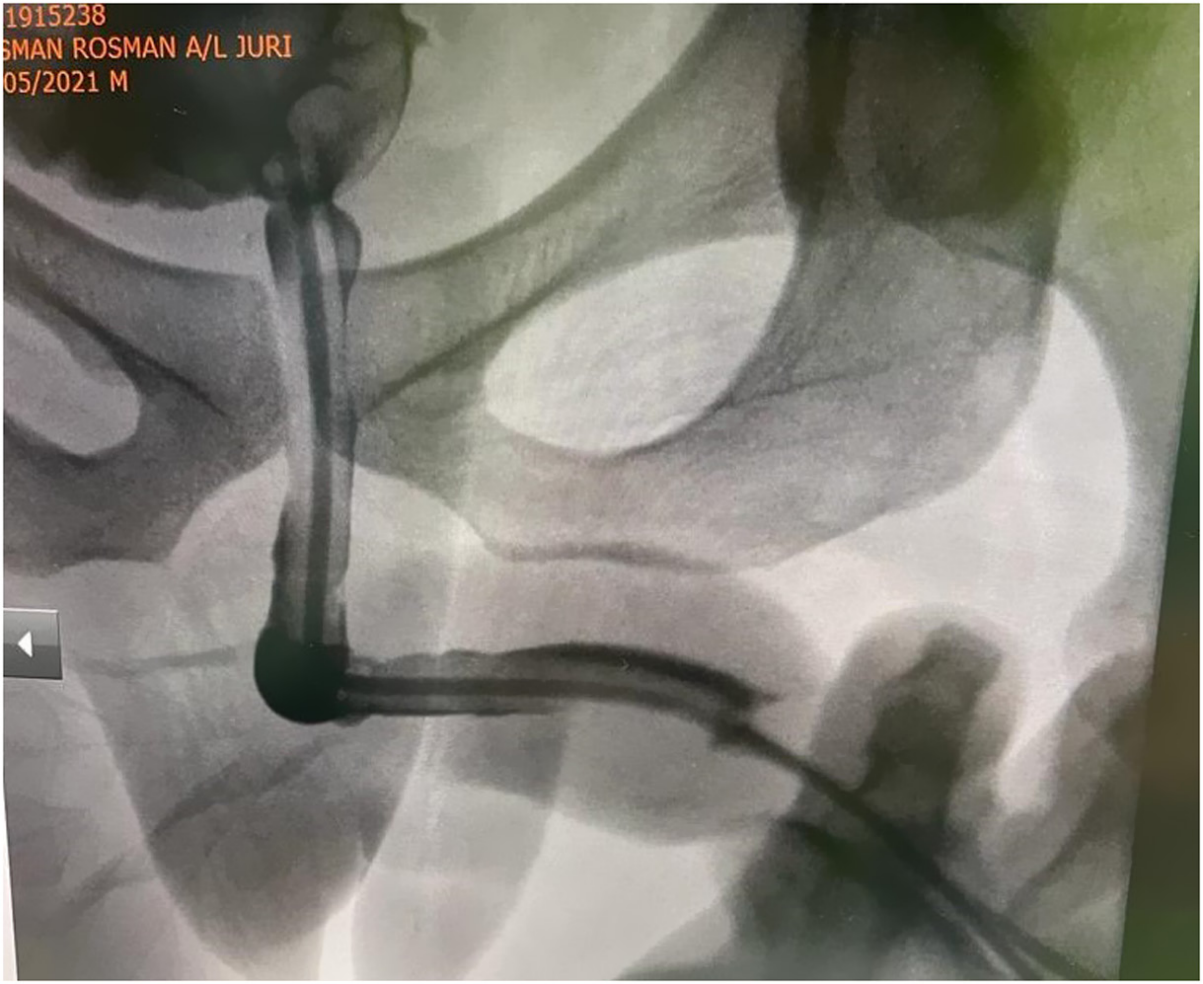
Pericathetogram postop showed intact urethral alignment.

Notably, immediate primary anastomotic urethroplasty was performed in our patients as there was minimal distortion of anatomy and little peri-urethral hematoma. There were clear opportunities to avoid the need for delayed, secondary procedures. During the repair, we needed minimal mobilization of the urethra and spatulated the urethra before performing an end-to-end anastomosis. Although delayed urethroplasty is a treatment option with a lower stricture rate, this technique is associated with a high incidence of incontinence.^8^ Notably, immediate primary anastomotic urethroplasty was performed in our patients. During the repair, we needed to mobilize and spatulate the urethra before performing an end-to-end anastomosis using 4-0 Vicryl sutures over a 16-French silicone catheter. The urethra was anastomosed using a parachute technique as shown in Figures 2 and 3. Both patients recovered well postoperatively with normal ascending urethrogram postoperatively as showed in Figure 4; however, one of the patients experienced stress incontinence, probably due to impaired sphincteric function because of direct injury to the arcus tendinous fascia surrounding the external sphincter. Both patients reported excellent outcome measures during the follow-up.

In cases of perineal trauma, a distended bladder is easily ruptured because of its high wall tension and impact during the accident. With the high incidence of urethral and bladder injuries, retrograde urethrography and cystography are the gold standard methods of investigation according to the EAU guidelines.^7^ Although extraperitoneal bladder injury can be managed conservatively, it is recommended that the bladder be explored and repaired in cases of extraperitoneal bladder injuries associated with pelvic fractures and anorectal injuries. Patients who underwent primary bladder repair were subjected to stress cystogram assessment 2 weeks postoperatively, and all of them had normal cystograms. 

The management protocol for anorectal injuries in adults consists of closure of the perineal wound after debridement and fecal diversion with or without presacral drainage. A study conducted by Haut et al^9^ showed that 62% of primary repairs with colostomy diversion in pediatric patients with penetrating anorectal injuries are safe. They recommended diversion colostomy in a group of patients with shock, multiple blood product transfusions, extensive perineal injuries with multiorgan involvement, severely contaminated wounds, or injuries caused by high-speed weapons. The decision of primary closure for anorectal injuries remains debatable in some studies for managing degloving perineal injuries.^10,11^ Theoretically, the impact of high amounts of force on the perineal region can be transmitted to the deep fascia and beyond. Therefore, we believe that prophylactic diversion colostomy leads to better wound healing as it reduced the complications of wound sepsis.

Kudsk and Hanna^2^ recommended jejunostomy and daily debridement and pulsatile irrigation for the first 3 days, in addition to our center’s management protocols. It was found that jejunostomy can establish early nutritional requirements and improve patients’ recovery. Additionally, they reported that daily debridement to eradicate the potential necrotic tissue significantly reduced postoperative sepsis.^2^ However, daily wound debridement would be associated with a higher risk of anesthesia, especially in elderly patients. We believe that early enteral feeding provides essential nutrients after major operations. Insertion of a jejunostomy tube is a good initiative; however, it is associated with the risk of tube dislocation or obstruction, intra-abdominal abscess, and enterocutaneous fistulas.^2^

Nutritional support plays an important role in patients’ postoperative recovery. Physiologically, metabolic and inflammatory hormones are secreted postoperatively. This “stress response” of the body leads to a catabolism state of hyperglycemia along with the breakdown of skeletal muscle to provide sufficient essential amino acids. Hence, early enteral feeding, particularly immunonutrition, should be established to improve recovery. In our center, we refer all postoperative patients with degloving wounds to dieticians to optimize nutrition and minimize the adverse effects of catabolism.

In the present case series, wound sepsis was the most common postoperative complication. Four of the 6 patients in this study had wound infections after primary closure of the wound. Radical wound debridement and the administration of broad-spectrum antibiotics are key steps for reducing the risk of sepsis. Moreover, prophylactic diversion colostomy helps to improve wound healing by preventing wound soiling, and all patients recovered well with conservative treatment. Our complication rates were similar to those of recent studies that reported pelvic sepsis as the main cause of late morbidity and mortality.^3^ Despite the favorable outcomes of the patients in this study, this report had a limited number of patients to provide significant feedback on the side effects and drawbacks of our surgical approaches.

In conclusion, a multidisciplinary team consisting of general surgeons, urologists, orthopedic surgeons, and plastic surgeons should be involved in managing complex perineal injuries. The implementation of aggressive resuscitation in trauma cases is important to minimize early mortality. Additionally, prompt diagnosis and immediate surgical intervention remain the standard treatments for complex perineal degloving injuries. Decisions regarding prophylactic diversion colostomy are important to minimize the risk of postoperative wound sepsis, and early urinary diversion and primary anastomotic urethroplasty are recommended for degloving perineal injuries with a transacted urethra. Our proposed management protocol that was described in this review appears to be safe and effective.

## Figures and Tables

**Table 1.** Degloving Perineal Injuries in 6 Cases

Patient	Etiology	Perineal Injury	Genitourinary	Colostomy	Pelvic Fracture	Complication	Sequelae
1	Motor vehicle accident	Avulsed buttock, rectum, and scrotum	Transacted bulbomembranous urethra	Yes	Yes	Wound sepsis	Anal and urinary incontinence, shorter penis length
2	Motor vehicle accident	Soft tissue, scrotum, and rectum	Urethral contusion	Yes	No	Wound sepsis	Anal incontinence
3	Motor vehicle accident	Soft tissue and scrotum	Urethral contusion	No	No	Nil	Nil
4	Motor vehicle accident	Soft tissue	Urethral stretch injury and bladder perforation	No	Yes	Hospital acquired pneumonia	Nil
5	Fall	Thigh, groin, and scrotum	Bladder perforation	No	No	Wound sepsis	Nil
6	Wild boar attack	Scrotum	Transacted penile urethra with breach at the corpus cavernosum	No	No	Nil	Rotated penis
